# Regulation of T-Cell Immune Responses by Pro-Resolving Lipid Mediators

**DOI:** 10.3389/fimmu.2021.768133

**Published:** 2021-11-16

**Authors:** Javier Perez-Hernandez, Valerio Chiurchiù, Sylvain Perruche, Sylvaine You

**Affiliations:** ^1^ Université de Paris, Institut Cochin, CNRS, Institut National de la Santé et de le Recherche Médicale (INSERM), Paris, France; ^2^ Departament of Nutrition and Health, Valencian International University (VIU), Valencia, Spain; ^3^ Institute of Translational Pharmacology, National Research Council, Rome, Italy; ^4^ Laboratory of Resolution of Neuroinflammation, European Center for Brain Research, Istituto di Ricovero e Cura a Carattere Scientifico (IRCCS) Santa Lucia Foundation, Rome, Italy; ^5^ Université de Bourgogne Franche-Comté, INSERM, Etablissement Français du Sang (EFS) Bourgogne-Franche Comté (BFC), Unité Mixte de Recherche (UMR)1098 Research on Interaction between Graft, Host and Tumor (RIGHT), Interactions Hôte Greffon-Tumeur/Ingénierie Cellulaire et Génique, Fédération Hospitalo-Universitaire Integrated Center for REsearch in inflammatory diseASes (InCREASe), Besançon, France; ^6^ MED’INN’Pharma, Besançon, France

**Keywords:** resolution, adaptive immunity, autoimmunity, T cell, therapy, specialized pro-resolving lipid mediators (SPMs), chronic inflammation

## Abstract

Both the initiation and the resolution of inflammatory responses are governed by the sequential activation, migration, and control/suppression of immune cells at the site of injury. Bioactive lipids play a major role in the fine-tuning of this dynamic process in a timely manner. During inflammation and its resolution, polymorphonuclear cells (PMNs) and macrophages switch from producing pro-inflammatory prostaglandins and leukotrienes to specialized pro-resolving lipid mediators (SPMs), namely, lipoxins, resolvins, protectins, and maresins, which are operative at the local level to limit further inflammation and tissue injury and restore homeostasis. Accumulating evidences expand now the role and actions of these lipid mediators from innate to adaptive immunity. In particular, SPMs have been shown to contribute to the control of chronic inflammation, and alterations in their production and/or function have been associated with the persistence of several pathological conditions, including autoimmunity, in human and experimental models. In this review, we focus on the impact of pro-resolving lipids on T cells through their ability to modulate T-cell responses. In particular, the effects of the different families of SPMs to restrain effector T-cell functions while promoting regulatory T cells will be reviewed, along with the underlying mechanisms. Furthermore, the emerging concept of SPMs as new biological markers for disease diagnostic and progression and as putative therapeutic tools to regulate the development and magnitude of inflammatory and autoimmune diseases is discussed.

## Introduction

The natural resolution of inflammation is a tightly controlled dynamic process that engages several molecular and cellular mediators to prevent excessive and/or chronic immune responses and tissue damage that may compromise organ function. Indeed, a dysregulation of this process has been incriminated in many inflammatory disorders (1–5). While the mechanisms regulating the onset of inflammation have been well characterized for almost a century and are mainly carried out by innate immune cells, which release pro-inflammatory lipids (i.e., eicosanoids: prostaglandins, leukotrienes, and thromboxanes) and cytokines/chemokines, the first endogenous mechanisms that terminate the inflammatory response have been identified exactly 20 years ago and now comprise over 25 lipid mediators derived from polyunsaturated fatty acids (PUFAs) and collectively termed specialized pro-resolving mediators (SPMs). The biosynthesis of all SPMs identified to date is initiated by the enzymatic addition of oxygen to four dietary PUFAs, namely, ω-6 arachidonic acid (AA), ω-3 eicosapentaenoic acid (EPA), ω-3 docosahexaenoic acid (DHA), and ω-3 docosapentaenoic acid (DPA), by means of the stereoselective and concerted action of the very same enzymes used for eicosanoid production, namely, the lipoxygenase (LOX) isozymes, the cyclooxygenase-2 (COX-2), and, to a minor extent, the cytochrome P450 (6).

**Figure 1:**
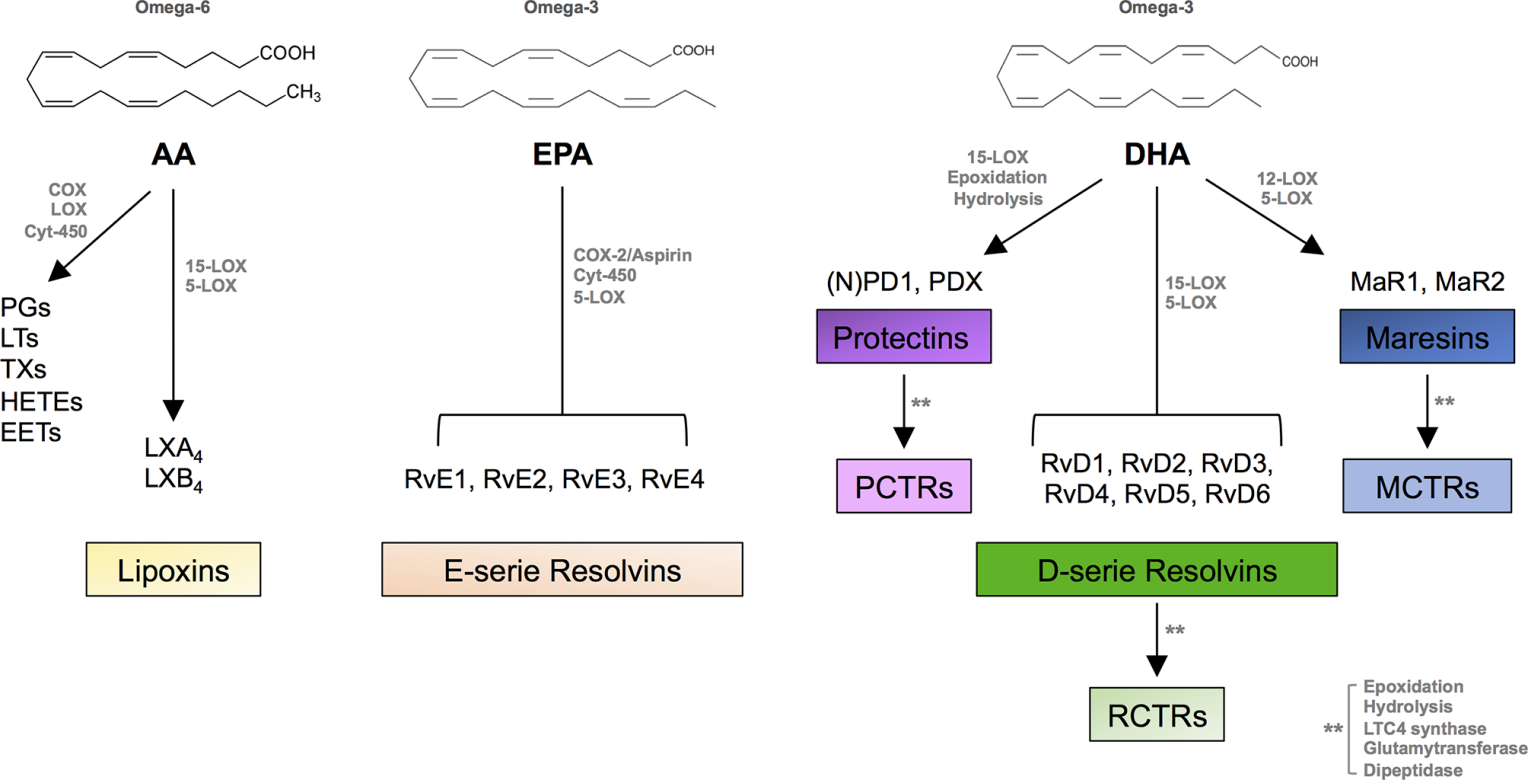
SPM biosynthesis. Chemical structures of AA, EPA, and DHA and outline of the individual families of the main pro-inflammatory eicosanoids and SPMs biosynthesized from these PUFAs. AA, arachidonic acid; COX, cyclooxygenase; LOX, lipoxygenase; Cyt, cytochrome; PGs, prostaglandins; LTs, leukotrienes; TXs, thromboxanes; EETs, epoxy eicosatrienoic acids; HETEs, hydroxy eicosatetraenoic acids; Rv, resolvin; PD, protectin; MaR, maresin; PCTR, protectin conjugates in tissue regeneration; RCTR, resolvin conjugates in tissue regeneration; MCTR, maresin conjugates in tissue regeneration.

SPMs include distinct families of bioactive lipids: AA-derived lipoxins (LXA_4_ and LXB_4_), EPA-derived E-series resolvins (RvE1-RvE4), DHA-derived D-series resolvins (RvD1-RvD6), protectins (PD1 and PDX), and maresins (MaR1-MaR2), together with their respective sulfide-conjugates in tissue regeneration (RCTR1-3, PCTR1-3, and MCTR1-3), DPA-derived 13-series resolvins (RvT1-RvT4), and RvD_n-3DPA_ (**Figure 1**). These lipid mediators, identified between 2001 and 2021 in the laboratory of Prof. Serhan in many tissues during acute inflammation (inflammatory exudates, plasma, brain, lymph nodes, etc.), are mostly produced locally by professional phagocytes [tissue-resident macrophages, recruited monocytes, immature dendritic cells (DCs)] and also by vascular endothelial cells and fibroblasts, to some extent. They act as “immunoresolvents,” i.e., immune-pharmacological agents of resolution (distinct from immunosuppressive agents), and induce (i) cessation of leukocyte infiltration and stimulation of nonphlogistic recruitment of mononuclear cells, (ii) macrophage-mediated phagocytosis of apoptotic polymorphonuclear cells (efferocytosis) and cellular debris, (iii) killing and clearance of pathogens, (iv) production of anti-inflammatory mediators while inhibiting secretion of pro-inflammatory cytokines and reactive oxygen species, (v) shortening time of resolution and activation of endogenous resolution programs, and (vi) promotion of tissue regeneration and healing (5–7).

SPMs trigger their pro-resolving signals *via* specific transmembrane G protein-coupled receptors (GPCRs) that display a variable level of redundancy and include FPR2 (or ALX), GPR32 (or DRV1), GPR18 (or DRV2), ChemR23 (or ERV), BLT1 (or LTB4R/GPR16), LGR6, GPR37, RORα, and GPR101 (8, 9). Of note, each SPM can act by activating different receptors, and each receptor is often engaged by several SPMs, which can also compete with pro-inflammatory lipids for binding. For instance, leukotriene B4 (LTB4) and RvE1/E2 are both ligands for BLT1 but exert opposite effects by respectively acting as an agonist (i.e., favoring neutrophil survival) or an antagonist (promoting neutrophils apoptosis) (10, 11).

The role of SPMs has been extensively explored in the context of inflammation-resolution operated by innate immune cells (mainly neutrophils and monocytes/macrophages) to resolve acute inflammatory diseases and infections (12, 13). However, accumulating evidence now pleads for an additional direct action of SPMs on the adaptive immune system and highlights their capacity to prevent the transition into chronic inflammation (5, 14, 15). Indeed, T lymphocytes express SPM receptors and are sensitive to omega-3- and omega-6-derived bioactive lipids, which, in the local microenvironment, can exert pro- or anti-inflammatory actions and thus influence T-cell fate and functions. More generally, it becomes increasingly clear that the mechanisms controlling T-cell responses and resolution of inflammation are tightly linked.

In this review, we will present in both human and mouse the evidence highlighting the role of SPMs, in comparison to their pro-inflammatory lipid counterparts, in the regulation of T-cell responses and T-cell-mediated immunity and autoimmunity. We will also discuss their putative usage as biomarkers of disease development or progression as well as therapeutic agents to restore tissue and immune homeostasis through their direct and/or indirect targeting of T cells.

## Pro-inflammatory Lipid Mediators and T Cells

The first evidence for a role and action of bioactive lipids on T cells has been documented in the early 2000s by studies uncovering the capacity of pro-inflammatory leukotrienes (LTs), especially of LTB4, to mediate effector T-cell (Teff) recruitment to inflammatory sites. Indeed, activated CD4^+^ and CD8^+^ Teff express the LTB4 high affinity receptor BLT1 but not naïve T cells (16, 17). LTB4 chemoattractant property was demonstrated in experimental models of peritonitis (16), asthma (18), transplantation (19), airway hyperresponsiveness (20, 21), and oxazolone (OXA)-induced contact dermatitis (22). T-cell trafficking into peripheral tissues was impaired in BTL1-deficient mice or in mice treated with a BLT1 antagonist, leading to the reduction of inflammation, graft rejection (19), and airway responsiveness (21). Adoptive transfer models using BLT1^+/+^ versus BLT1^-/-^ CD8^+^ T cells further strengthened the key role of the LTB4-BLT1 pathway in the migration of Teff to inflamed tissues or tumoral niches and the development of immune responses (16, 20, 21, 23). *In vitro* studies on mouse and human T cells confirmed the capacity of LTB4 to promote effector function by increasing Th1 (IFN-γ), Th2 (IL-4), and Th17 (IL-17) cell migration and responses while decreasing Foxp3^+^ regulatory T cells (Tregs) in polarization assays (17, 24, 25). LTB4 was also shown to induce the differentiation of T follicular helper (Tfh) cells from naïve T cells, which activate naïve B cells to form germinal centers (26).

**Figure 2:**
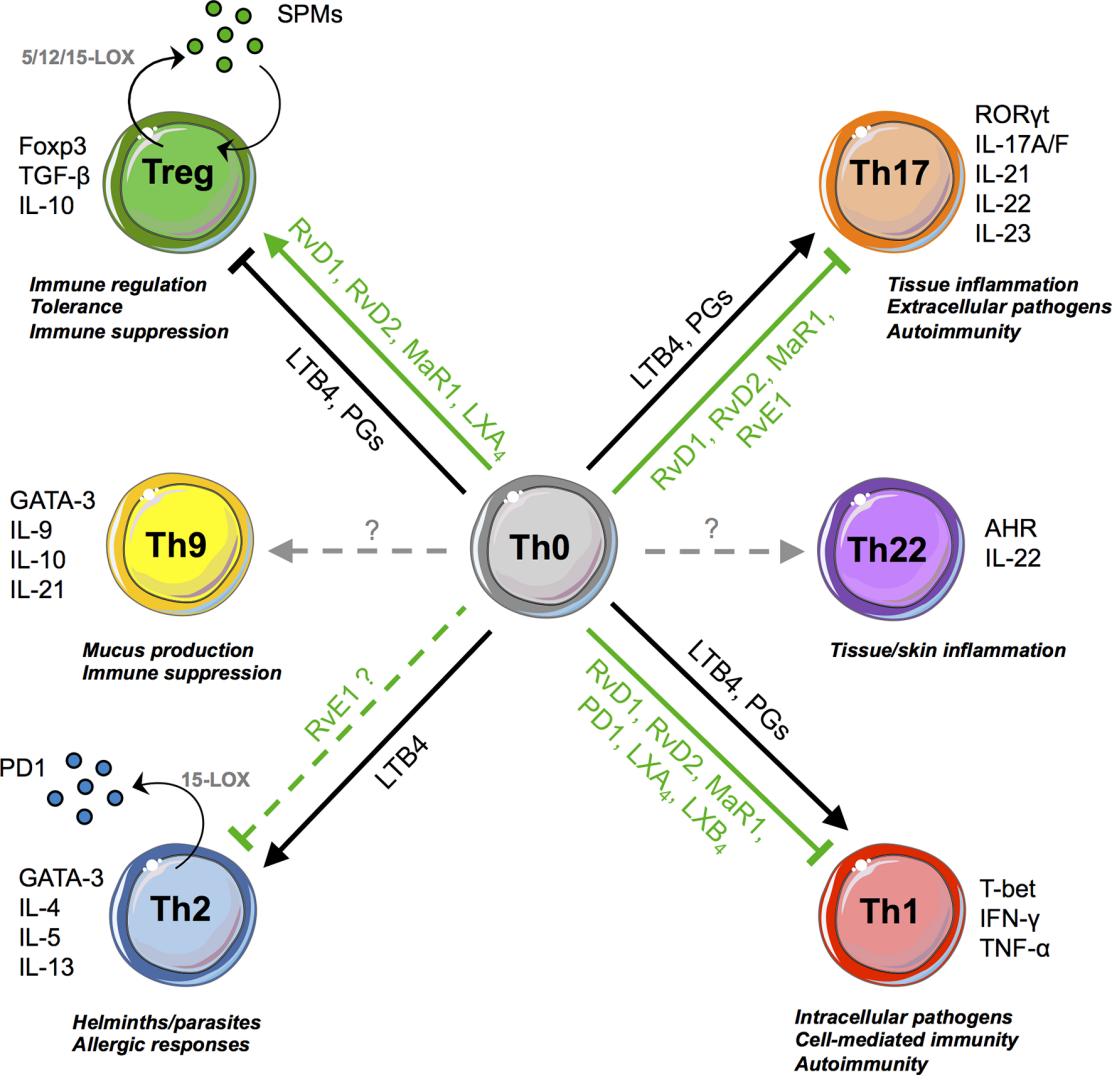
SPMs and T-cell responses. SPMs and pro-inflammatory lipids promote or inhibit, in opposite ways, T-cell differentiation (from Th0 precursors to Tregs, Th1, Th2, Th17). Th2 and Treg cells are also able to biosynthesize few SPMs, such as 17R-RvD3, RvD4, MaRn-3DPA, and PD1, respectively.

Prostaglandins (PGs) also affect T-cell responses through binding to the E-prostanoid receptors (EP)1-4 (27). Notably, PGE2 boosts and expands Th1 and Th17 cells, which express EP2 and EP4, while inhibiting Th2 cells (28). T cells deficient for EP4 are insensitive to PGE2 and downregulate IL-12 and IFN-γ receptor expression, reducing their *in vivo* pathogenicity (28). Similarly, administration of EP antagonists impaired the development of experimental arthritis, psoriasis, and multiple sclerosis through the inhibition of Th1 and Th17 responses (29–31). These pro-inflammatory effects are in line with the capacity of PGE2 to dampen the differentiation of type 1 regulatory T cells (Tr1) (32) and Foxp3^+^ Tregs (33), although some contradictory results were reported (34). Overall, both LTs and PGs, being central in the perpetuation of inflammatory signals that are at the basis of the transition from acute to chronic inflammation, act as “cytokine amplifiers” by stimulating almost all Teff subsets (**Figure 2**).

## Immunomodulatory Role of SPMs on Pathogenic T Cells

T-cell activation and differentiation into T helper (i.e., Th1, Th2, Th17, Th22 cells) or cytotoxic subsets is instrumental to fight pathogens. They must, however, be controlled and limited in space and time to avoid T-cell hyperactivation, a hallmark of chronic inflammation and autoimmunity. Accordingly, an increasing number of recent studies in mice and humans now document the capacity of several families of SPMs to inhibit pathogenic T cells (**Figure 2**), thereby contributing to the resolution of inflammation.

Initial studies on the effect of SPMs on T cells came from lipoxins, whereby LXA4 and LXB4 inhibited ERK-dependent TNF-α secretion from peripheral T cells stimulated with anti-CD3 antibody (35). These effects were mediated by the LXA4 receptor FPR2/ALX, expressed at a higher level on activated CD25^+^ and memory CD45RO^+^CD4^+^ T cells, with respect to their CD25^-^ and naïve CD45RA^+^CD4^+^ counterparts (35, 36). Accordingly, treatment with LXA4 inhibited Th1 and Th17 activation, as observed *in vivo* in the cervical draining lymph nodes of mice with dry eye disease (37), and *in vitro* using peripheral blood from healthy donors (38). LXA4 was also reported to differentiate naïve CD4^+^ T cells from tonsils and peripheral blood into Tfh cells in an FPR2-dependent manner (26).

The evidence that the other SPM families were capable of modulating T-cell responses was first unveiled through indirect studies showing reduced T-cell migration into target organs and decreased production of inflammatory cytokines after SPM *in vivo* administration in various experimental models of inflammation (detailed in the last section and **Table 1**). The capacity of SPMs to directly impact T-cell response was demonstrated by Chiurchiù and colleagues, who extended these observations to RvD1, RvD2, MaR1, and aspirin-triggered RvD3. Indeed, these SPMs were able to reduce the pro-inflammatory activity of both human CD4^+^ and CD8^+^ T cells upon stimulation of their T-cell receptor, to inhibit cytokine production by circulating Th1 and Th17 cells, and to prevent Th1 and Th17 commitment of naïve CD4^+^ T cells, without, however, impacting T-cell viability and proliferative capacity (52). These findings were also supported by the *in vivo* evidence that mice deficient for elongase 2, the key enzyme involved in the biosynthesis of DHA, the precursor of D-resolvins, protectins, and maresins, showed increased percentages of Th1 and Th17 cells, which were reduced upon dietary supplementation with DHA or *in vivo* treatment with RvD1 (52). Of note, the capacity of SPMs to prevent Th17 polarization was also observed in patients with inflammatory diseases such as rheumatoid arthritis or systemic lupus erythematosus (59, 63). Interestingly, RvD1, RvD2, and MaR1 were not capable to modulate IL-4 production and Th2-cell development *in vitro* (which may, however, not reflect *in vivo* processes) (52). These results are in line with studies reporting that, in Th2-driven pathologies and mouse models, DHA-derived SPMs like RvD1 and PD1 do not affect IL-4 release and might ameliorate clinical outcome by acting on different cellular targets (64, 65).

**Table 1:** *In vivo* treatment with SPMs and impact on T-cell subsets and functions.

SPM	Disease/*Model*	Treatment	Therapeutic efficacy and MOA	Ref.
RvE1	Allergic airway-inflammation/*mouse*	5,000 ng/kg/day for 3 days (iv)	Airway inflammation resolution:↓ IL-23 and IL-6 and cell infiltrate within the bronchoalveolar lavage fluid.↑ Th1/Th17 ratio.	(39)
	HSV-induced stromal keratitis (SK)/*mouse*	60,000 ng/kg/day for 8 days(topical, eye)	↓ SK severity↓ cornea influx of neutrophils, Th1 and Th17 cells, IFN-γ, and IL-6.	(40)
	Imiquimod (IMQ) challenge-induced psoriasis/*mouse*	8,000 ng/kg/day (iv)	↓ psoriasis severitySuppression of IL-23-producing DCs and γδ T cells.	(41)
	Corneal allograft/*mouse*	50,000 ng/kg at day 0 and 7 (subconjunctival)	↑ graft survival↓ Th1 and Th17 infiltration and edema into the graft and draining lymph nodes	(42)
	Ligature-induced periodontitis/*mouse*	140 ng/kg/day for 10 days (topical)	↓ bone loss↓ T-cell infiltrate and preservation of Tregs.	(43)
	Hypersensitivity skin model/*mouse*	10,000 ng/kg/day at day -1, 0, and 5 (iv)	↓ ear swelling↓ IFN-γ-producing CD8^+^ T cells in the skin	(44)
	Femoral artery wire injury/*mouse*	8,000 ng/kg/day (ip) (2 days before surgery)	↓ T-cell recruitment to perivascular areas.↓ IFN-γ and IL-2 mRNA levels in injured arteries.	(45)
RvD1	Endotoxin-induced uveitis/*rat*	10 to 1,000 ng/kg (iv/intravitreal)	↓ uveitis.↓ neutrophils, T and B cells, M1 macrophages infiltration↓ TNF-α, CXCL8, and RANTES in the eye.	(46, 47)
	Experimental autoimmune encephalomyelitis/*mouse*	5,000 ng/kg/day for 40 days (oral)or during 15 days starting at day 7 (ip)	↓ disease severity↓ autoreactive T cell infiltration	(48)
	OVA-induced allergic eye disease (AED)/*mouse*	1,000 ng/kg/day for 7 days (topical)	↓ AED score↓ conjunctival immune cells (except macrophages).	(49)
	Experimental autoimmune neuritis (EAN)/*rat*	5,000 ng/kg/day for 12 days (ip)	Enhanced EAN recovery↓ effector T cells.↑ Tregs and IL-10, TGF-β.	(50)
	Ischemia/reperfusion-induced acute kidney injury/*mouse*	5,000 ng/kg/day for 3 days (iv)	↑ Tregs and alleviated renal tubular injury.↓ serum levels of IFN-γ, TNF-α, and IL-6 in a ALX/FPR2-dependent pathway.	(51)
	DHA deficiency (Elovl2^−/−^)/*mouse*	5,000 ng/kg/day with 50 µg of anti-CD3 (ip)	↓ IFN-γ and IL-17 by peripheral blood CD4^+^ T cells	(52)
RvD1/RvE1	Concanavalin A-induced hepatitis/*mouse*	10,000 ng/kg/day (iv)	↓ liver injury↓ CD4^+^ and CD8^+^ T-cell liver infiltration, inflammatory cytokines, and NF-κB/AP-1 activity.	(53)
RvD2	Porphyromonas gingivalis-induced experimental periodontitis/*mouse*	25,000 ng for 3 days plus 5,000 ng for 6 days (over 2 weeks) (ip)	Prevent alveolar bone loss↓Th1 priming and chronic IFN-γ secretion.↑ pro-resolving macrophages in the gingiva.↓Tregs.	(54)
PD1	Zymosan A-induced peritonitis/*mouse*	5,000 ng/kg, 2 h prior to challenge (iv)	↓ T-cell infiltration in the peritoneum	(55)
	IMQ-induced psoriasis/*mouse*	10–1,000 ng/kg/day for 7 days (sc)	↓ psoriasis severity↓ inflammatory cytokines in lesion and serum↓ Th1/Th17 cells in spleen	(56)
	HSV-induced stromal keratitis (SK)/*mouse*	15,000 ng/kg twice/day for 10 days(topical, eye)	↓ SK severity↓ infiltration of neutrophils and pathogenic CD4^+^ T cells↓ inflammatory cytokines, chemokines, and angiogenic factors in the cornea	(57)
MaR1	OVA-induced allergic inflammation/*mouse*	50 ng/kg/day for 4 days (iv)	↑ Tregs↓ IL-13 secretion by ILC2 in the bronchoalveolar lavage fluid	(58)
	Collagen-induced arthritis/*mouse*	5,000 ng/kg/day for 16 days (iv)	↓ arthritis severity↑ Treg/Th17 ratio (draining lymph node)	(59)
	IMQ-induced psoriasis/*mouse*	8,000 ng/kg/day for 5 days (topical)	↓ skin inflammation↓ IL-17-producing CD4^+^ and γδ T cells in the skin.	(60)
	Spontaneous colitis/*IL-10^-/-^ mouse*	50 ng/kg/day for 14 days (ip)	↓ CD4^+^ T cells in colon *lamina propia*↓ TNF-α, IFN-γ, IL-6, and IL-17 levels in colon	(61)
LXA4	Autoimmune dry eye/*mouse*	5,000–50,000 ng/kg/day for 10 days(topical/systemic)	↓ Th1 and Th17 cells in draining lymph nodes↑ Treg	(37)
	Experimental autoimmune encephalomyelitis (EAE)/*mouse*	5,000 ng/kg/day, daily (ip)	↓ EAE severity↓ Th1 and Th17 cell infiltration into the central nervous system↓ levels of pro-inflammatory lipids in spinal cord fluid.	(38)
	Experimental autoimmune uveitis (EAU)/*mouse*	40,000 ng/kg (mouse; ip)/day, daily	↓ EAU severity↓ Th1 and Th17 cell infiltration↓CD4^+^ T cell glycolytic responses and IFN-γ production	(62)

CD, cluster of differentiation; DC, dendritic cell; HSV, herpes simplex virus; IFN, interferon; IL, interleukin; ILC, innate lymphoid cells; ip, intraperitoneal; iv, intravenous; LXA4, lipoxin A4; MaR1, maresin 1; MOA, mode of action; OVA, ovalbumin; PD1, protectin 1; RANTES, regulated upon activation, normal T cell expressed and secreted; RvD1, resolvin D1; RvD2, resolvin D2; RvE1, resolvin E1; sc, subcutaneous; TCR, T-cell receptor; Th, T helper; TNF, transforming necrosis factor; Treg, regulatory CD4^+^ T cell.

In contrast, RvE1, which is derived from EPA, was reported to promote resolution of asthmatic airway inflammation and atopic dermatitis by reducing Th2 cytokines; however, evidence of a direct T-cell targeting is missing (66–68). Although data supporting the suppressive effect of RvE1 on Th1, Th2, and Th17 cells are mostly indirect, obtained either upon *in vivo* administration or *in vitro* stimulation of dendritic cells (39, 40, 42, 66–71), a very recent study showed that this bioactive lipid can directly impact Th17 development by inhibiting their IL-6 and TGF-β-induced polarization (72). The effect of SPMs on other T helper subsets like Th22 and Th9, either direct or indirect, is still unknown.

SPM-mediated modulation of T-cell fate and function is associated with the expression of several SPM receptors, including ALX/FPR2, GPR32, GPR18, BLT1, and ChemR23, with higher levels reported on activated and effector cells (albeit at significantly lower levels compared to innate immune cells), indicating that activated T cells are more responsive to SPMs in general (35, 36, 52). However, clear data on their expression and signaling pathway in the different T-cell subsets remain currently very limited and deserve further investigations. Accordingly, human Th1 express higher mRNA levels of ALX/FPR2 and also higher protein levels of GPR32 compared to Th0 (52), while Th17 express higher protein levels of both GPR32 and ChemR23 (52, 72). These receptors account for the reported effects of RvD1 and RvE1 in reducing Th1 and Th17 responses. The role of the other SPM-binding receptors in mediating the effects of RvD2, PD1, and MaR1 remains to be explored. This may be related to their more recent discovery, such as GPR37 that was identified as the PD1 receptor in 2018 and investigated only in macrophages (73) or LGR6, the surface receptor for MaR1, identified in 2019 and mainly expressed in neutrophils and macrophages, with little expression on total CD3^+^ T cells (74). Thus, additional thorough studies are necessary at the systemic and tissue levels to better decipher the expression profile and activity of SPM receptors on T cells, which may account for their activation, migration, and control.

Lastly, while it is now well documented that T cells can respond to several SPMs, little information is known about their capacity to produce pro-resolving lipids. As yet, only one study reported that human Th2-polarized peripheral blood mononuclear cells (PBMCs) with IL-4, but not IL-12/IFNγ-polarized Th1 cells, produced PD1 in a 15-LOX-dependent manner (55). However, due to likely cellular contaminants in the experimental setting using total PBMC and not highly purified naïve CD4 T cells, the possibility that other IL-4-responding cell types (i.e., monocytes and dendritic cells) were the source of PD1 cannot be ruled out.

## Impact of SPMs on Regulatory T Cells

Among all T-cell subsets, Tregs are arguably the one that fits the most with the concept of resolution of inflammation due to their crucial role in maintaining immune equilibrium and homeostasis (75). They not only play a vital role in the prevention of autoimmunity and the maintenance of self-tolerance but have also been shown to promote macrophage pro-resolving functions, i.e., efferocytosis through an IL-13/IL-10/Vav1 pathway, expression of SPM receptors, and SPM production in target tissues (76, 77). Of note, recent studies suggest that the abundance and function of Tregs change during inflammation and that the pool of peripheral Tregs is diverse and can derive from other pathogenic T helper cells; for instance, Th17 can transdifferentiate into Tregs during the resolution of inflammation (78).

Despite the key role of Tregs in regulating immune and tissue homeostasis, the experimental evidence reporting the impact of SPMs on these tolerogenic T cells dates back only to 2015, first with the *in vitro* demonstration of the direct effect of MaR1 in amplifying the TGF-β-induced *de novo* generation of Foxp3^+^ Tregs (iTregs) (58), and second with the *in vivo* observation of an increased frequency of Foxp3-expressing T cells after LXA_4_ treatment (37). These findings were extended to RvD1, RvD2, and MaR1 in the human setting, with iTregs showing increased CTLA-4 expression, IL-10 release, and suppressive capacity (52). However, although mice genetically unable to produce SPMs (Elovl2^-/-^ mice) displayed decreased proportion of Foxp3^+^ Tregs, none of the tested SPMs was capable, *per se*, to induce Foxp3 expression and convert naïve CD4^+^ T cells into Tregs without the required TGF-β-enriched polarizing milieu (52).

The positive impact of SPMs on Foxp3^+^ Tregs was further corroborated *in vivo* upon administration of SPMs (mainly RvD1, LXA4, and MaR1) in several experimental models of chronic inflammatory or neurodegenerative diseases, although the enhanced Treg proportion observed in these contexts may be due to both direct and indirect effect on T cells (50, 51, 59, 63, 79) (**Table 1**). Of note, a majority of these studies also reported an improved Treg/Th17-Th1 balance in the target tissue or draining lymph nodes, associated with SPM therapeutic efficacy. Concerning PD1, its *in vivo* administration has recently been associated with increased frequency of IL-10-producing CD4^+^ T cells in the colon, suggesting an impact on Tr1 cells (80). Yet, no evidence has been reported for a role of this DHA-derived SPM on Foxp3^+^ Tregs.

Only two studies have examined which receptor might be involved in mediating the SPM-induced Treg generation. Both studies focused on RvD1 and, by using neutralizing antibodies, they demonstrated that GPR32 was responsible for the Foxp3^+^ Treg differentiation in humans (52), while ALX/FPR2 was at play in mice (51).

Interestingly, a recent work reported that human and mouse Foxp3^+^ Tregs express the 5/12/15-lipoxygenases and are able to biosynthetize several SPMs from all four major bioactive metabolomes, but only 17R-RvD3, RvD4, and MaRn-3DPA were significantly produced at a higher amount compared to naïve CD4^+^ T cells (81). Genetic ablation of ALOX15 in Tregs decreased Foxp3 expression and altered their transcriptional and metabolic programs, resulting in impaired suppressive function and increased effector pathways. Also, Foxp3 binding elements were identified in the ALOX15 promoter region, allowing a direct positive regulation of ALOX15 expression by Foxp3 (81).

Collectively, these findings demonstrate that SPMs can target different T-cell subsets to modulate their development and functions, and emphasize the pro-resolving and homeostatic actions of SPMs (**Figure 2**). They also pinpoint that mechanisms driving immune regulation and resolution of inflammation are complementary and interconnected, which supports the potential of new therapeutic opportunities. Additionally, SPMs may be produced not only by innate immune cells but also by T cells, possibly exerting autocrine and paracrine anti-inflammatory actions, ultimately contributing to immune regulation.

## Defective Pro-resolving Pathways Associated With Pathogenic T-Cell Responses

Chronic diseases are characterized by excessive inflammation and impairment of natural resolution mechanisms. Bioactive lipids are implicated in the pathologic processes, with an unbalanced production of inflammatory over pro-resolving lipids, driving the aberrant recruitment and activation of immune cells, including effector T lymphocytes, leading to tissue damage and ultimately disease symptoms (5). Pathogenic T-cell responses have been associated with defective resolution pathways and disease progression. However, so far, few studies have investigated and demonstrated a direct link between a deficient SPM pathway and exacerbated T-cell reactivity.

In experimental models of autoimmune dry eye disease or autoimmune neuritis (mimicking human inflammatory demyelinating polyradiculoneuropathy), reduced RvD1 or LXA4 amounts correlated with decreased proportion of Foxp3^+^ Tregs in the inflamed tissues, respectively (37, 50). In humans, the BLT1 receptor was found upregulated on T cells isolated from the airways of patients presenting obliterative bronchiolitis after lung transplantation (19), or from the blood of asthmatic patients (82). These data suggest an increased T-cell sensitivity to pro-inflammatory LTB4 and a critical role of BLT1 in T-cell recruitment into target organs as evidenced in animal models. Conversely, T cells from patients with chronic heart failure expressed reduced levels of GPR32 as compared to healthy subjects, correlating with an impaired responsiveness of CD4^+^ and CD8^+^ T cells to RvD1 and RvD2 (assessed by the inhibition of inflammatory cytokine production) (83). In the context of multiple sclerosis (MS), ALX/FPR2, GPR32, GPR18, and ChemR23 were differentially expressed on PBMCs according to disease activity, with decreased mRNA levels in patients with progressive MS as compared to patients presenting relapsing or remitting MS (84). Of note, while BLT1 showed similar expression in all donor groups, these SPM receptors were globally upregulated in relapsing or remitting MS compared to healthy donors, which may reflect the engagement of resolution mechanisms in immune cells, including T cells, in response to inflammation and autoimmunity. Lastly, a recent study showed that LXA4 modulated *in vitro* activation, TNF-α, IFN-γ, IL-17 production, and transendothelial migration capacity of CD4^+^ and CD8^+^ T cells from both healthy donors and patients with relapsing–remitting MS (38). However, we have to keep in mind that these analyses were performed on circulating T cells, not on tissue-infiltrating T cells, which may exhibit distinct functional characteristics and behavior influenced by the local inflammatory/resolving lipid balance.

Of interest, in the last years, lipidomic analysis of plasma or serum recovered from patients with chronic inflammatory disorders implicating pathogenic T-cell responses constantly showed decreased SPM concentrations at the time of active disease, compared with healthy and/or inactive disease status. This was reported for MaR1 and PD1 in rheumatoid arthritis (RA) (59, 85) and for RvD1 in SLE, MS, and chronic heart failure (63, 83, 84). Lower levels of RvD3, RvD4, and RvE3 were also observed in a small cohort of stage III RA patients, with concomitant increase in inflammatory TBX2 (86). In some instances, SPM abundance negatively correlated with disease severity (63, 84, 85). Similarly, patients with leprosy and acute Th1-mediated inflammatory episodes showed reduced plasma levels of RvD1 as compared to patients without Th1 hyperreactivity (87). In most cases, opposite patterns were observed for arachidonic acid-derived inflammatory lipids (LTB4, PGs), which were found in higher abundance at the time of progressive disease.

However, available information is overall limited, and caution should be brought on the interpretation of these results. Indeed, depending on the clinical context, higher levels of plasmatic SPMs can be detected in patients presenting inactive/controlled inflammatory and autoimmune pathologies as compared to healthy individuals, which may reflect perturbed resolution processes related to an attempt to counteract inflammation. In addition, systemic levels of bioactive lipids may not coincide with tissue abundance, and a decrease in the SPM level could result from higher consumption, reduced production, or both conditions. Lastly, expression of SPM receptors on T cells and their responsiveness to SPMs have so far been explored in few pathological contexts. These investigations need to be extended to additional immune disorders to better understand the impact of resolutive pathways on effector and regulatory T-cell subsets and to identify putative failures at the systemic and tissue levels, which may contribute to the defective immune regulation leading to disease progression.

Thus, these findings call for further deciphering, in mice and humans, the impact of resolution mechanisms and the SPM pathway on adaptive immunity, in particular their role in the dysregulated effector/regulatory T-cell responses responsible for the progression and chronicity of immune and autoimmune diseases.

## *In Vivo* Therapeutic Efficacy of SPMs Through T-Cell Reprogramming

**Figure 3:**
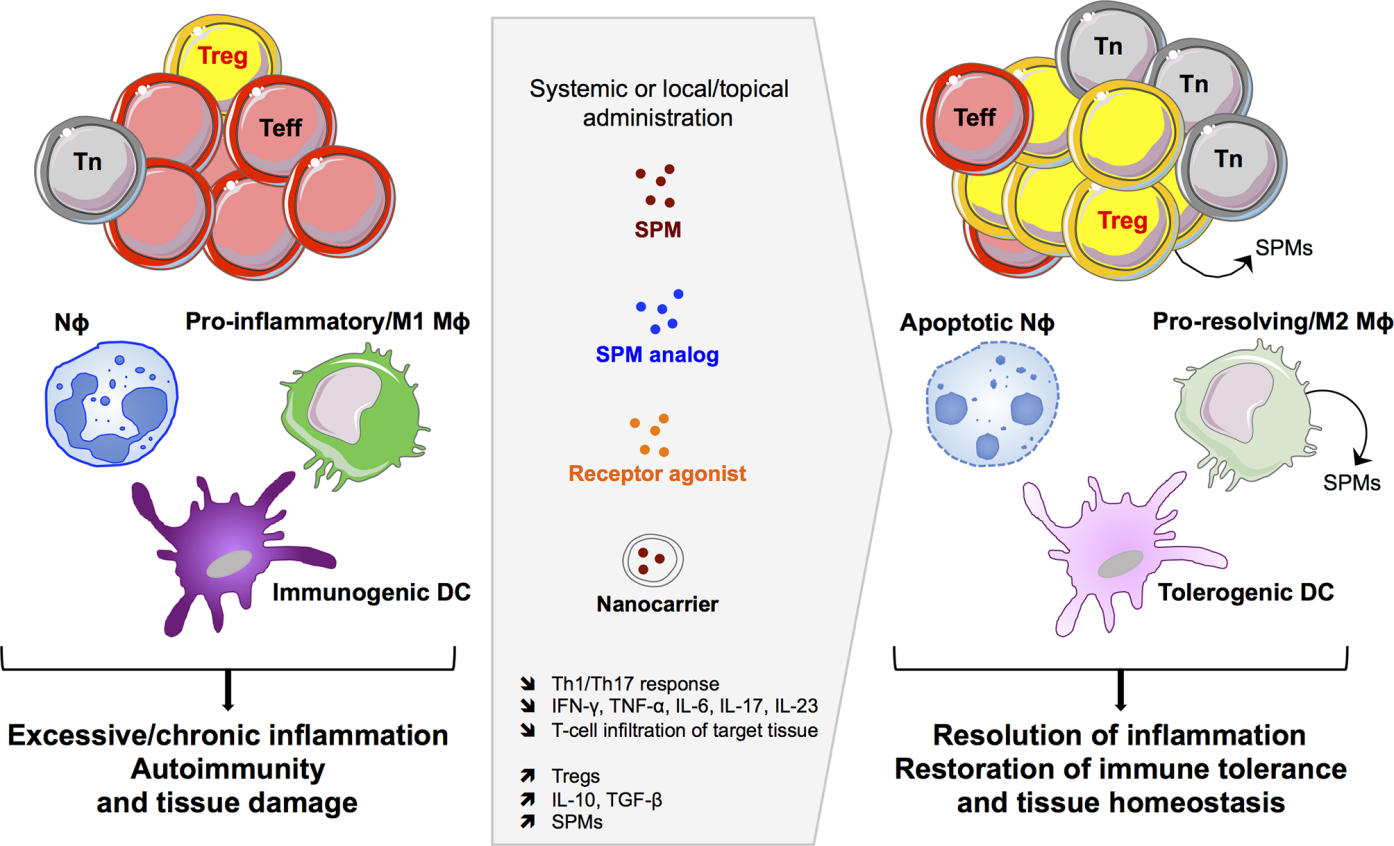
SPM-based therapeutic opportunities. Targeting the SPM pathway can impact innate and adaptive immune cells to induce efficient resolution of excessive inflammatory responses and to restore immune tolerance and tissue homeostasis. Nϕ, neutrophil; Mϕ, macrophage; DC, dendritic cell; Teff, effector T cell; Tn, naïve T cell; Treg, regulatory T cell.

The potent pro-resolving properties of SPMs have encouraged their therapeutic application in various experimental models of inflammation (**Table 1** and **Figure 3**). Particularly, RvD1 and RvE1, but also RvD2, MaR1, PDX, and LAX4, have been tested in a number of pathological contexts presenting resolution defects accentuated by effector T-cell infiltration and activity in target organs. The protocols used were very variable depending on the model: SPMs were administered through systemic (intravenous, intraperitoneal) or local (topical application on the eye, intravitreal, subconjunctival) routes, at an average dose of 5 μg/kg, i.e., approximately 100 ng/mouse (ranging from 20 to 500 ng/injection), with single or repeated injections over several weeks.

Overall, SPM treatment reduced the severity of inflammatory diseases (in most cases experimentally induced in animal models), marked by decreased tissue damage, immune cell infiltration, and inflammatory cytokines. Indeed, Th1 and Th17 cells were repeatedly found in lower numbers in target organs and draining lymph nodes, together with less *in situ* expression (mRNA and/or protein) of IFN-γ, TNF-α, IL-17, IL-6, and IL-1β in the context of endotoxic uveitis (46, 47), concanavalin-A-induced hepatitis (53), sepsis-induced lung injury (88), stromal keratitis (herpes simplex virus-induced ocular inflammatory lesions) (40, 57, 89), periodontitis (43, 54), and rheumatoid arthritis (59, 85). Similar positive outcomes were observed in allergic manifestations such as chronic allergic eye disease (49), allergic airway inflammation (39), and psoriatic dermatitis (41). SPM therapy has been explored in few autoimmune diseases, i.e., RvD1 in Sjögren’s syndrome (90) and SLE (63), and LXA4 in experimental autoimmune uveitis (EAU) (62) and encephalomyelitis (EAE) (38), with here again the amelioration of symptoms and reduction of pathogenic T-cell trafficking to target tissues and inflamed responses. Of note, the metabolic program of CD4^+^ T cells was found modulated in LXA4-teated mice, marked by decreased glycolytic responses associated with reduced IFN-γ production (62). Lastly, RvE1 has been applied in a model of corneal transplantation, promoting graft survival associated with diminished Th1 and Th17 CD4^+^ T cells and inflammatory cytokines in the allogeneic transplant (42, 91).

In some studies, the decrease in effector T lymphocytes observed after SPM treatment was paralleled by an increase in Fox3^+^ Treg frequency and associated immunomodulatory cytokines such as IL-10 and TGF-β (50, 51, 54, 59, 63, 85). Administration of SD-208, a TGF-β receptor antagonist, reversed the RvD1 therapeutic effect in autoimmune neuritis, showing the closed relationship between these two modulatory pathways (50). The cross-talk between SPMs and Tregs was highlighted in a model of ischemia/reperfusion-induced acute kidney injury (IRI-AKI mice) by the abrogation of RvD1 therapeutic effect after the administration of the anti-CD25 PC61 monoclonal antibody, which preferentially depletes Tregs (51). Furthermore, the beneficial effect of RvD1 on Treg frequency was suppressed after *in vivo* blockade of FPR2 with a specific antagonist (Boc-1), leading to severe renal tubular injury (51).

Therefore, these experimental data support SPM-based therapy as a promising avenue for the treatment of a wide range of T-cell-mediated immune and autoimmune diseases. Especially, the *in vivo* therapeutic efficacy of SPMs and associated dampening of effector T-cell responses can benefit from both a direct effect on T cells and an indirect action through antigen-presenting cells (APCs: DCs, macrophages), expressing SPM receptors, as SPMs downregulate APC activation, expression of costimulatory molecules, and secretion of inflammatory cytokines, which subsequently prime effector T cells (72).

In terms of clinical application, a pilot study has been launched in patients undergoing total knee arthroplasty to investigate the impact of preoperative supplementation with Lipinova^®^ (Solutex), described as a concentrate of EPA, DHA, and SPMs (NCT03434236). However, the clinical use of SPMs as therapeutic agents is impeded by their metabolically labile and unstable state, restricted bioavailability, and rapid clearance, and thus calls for the development of SPM synthetic analogs displaying pharmacokinetic properties in compliance with *in vivo* usage (92, 93). In this line, a RvE1 mimetic, RX-10045, has been tested as an ophthalmic solution for dry eye symptoms (NCT00799552), allergic conjunctivitis (NCT01639846), and in patients undergoing cataract surgery (NCT02329743). Promising results were recently reported in a phase 1 trial evaluating the safety and efficacy of BLXA4, a LXA4 analog, to treat periodontitis (NCT02342691): daily rinsing with mouthwash containing BLXA4 reduced gingival inflammation, and this was associated with a shift in serum lipid mediators toward a pro-resolving profile (94).

The development of SPM receptor agonists is also in the pipeline, as recently documented by the study of Trilleaud et al. who tested a new anti-ChemR23 agonist antibody in models of acute and chronic colitis (induced by DSS or adoptive transfer of CD4^+^CD45RB^high^ T cells, respectively) and inflammation-driven cancers (95). This antibody was able to activate the pro-resolving ChemR23 pathway and trigger the resolution of inflammatory responses.

## Conclusion

SPMs display a wide range of anti-inflammatory actions, which are increasingly explored in adaptive immunity, notably through their ability to bind several GPCRs expressed by T cells, to regulate T-cell functions, and to trigger the resolution of inflammatory and immune-mediated diseases in animal models. Of note, a number of studies also report SPM activity on humoral responses, with, however, some discrepant results, as RvD1 increased the production of IgM and IgG (96) while decreasing the secretion of IgE by activated B lymphocytes (97, 98). IgM and IgG production and antigen-specific memory B-cell responses were similarly reduced by LXA4 (99). Building on the potent immunoresolvent properties of SPMs and their multiple targets, these findings open a new field of investigations that may provide a better understanding of the physiological regulation of adaptive immune response and its failure in pathophysiological contexts. SPM lipidomic analyses are indeed performed in the frame of several clinical studies as a read-out of disease diagnosis and progression, inflammatory profile [for instance in COVID19 patients (100)], as well as response to therapeutic interventions (NCT04698291, NCT04452942, NCT04697719, NCT01865448, NCT04308889, NCT04377334, and NCT02719665) (101).

In addition, the pathways governing immune regulation and resolution programs are interrelated and provide the mechanistic rationale for developing SPM-based therapeutic strategies that can be beneficial for the treatment of chronic inflammatory disorders, autoimmune diseases, and organ transplantation, notably through the control of effector T cells and boost of Tregs. This is reinforced by the positive outcomes observed in various pathological settings after dietary intervention using SPM precursors, DHA and EPA, or omega-3 lipids, which enhance SPM levels and help in controlling excessive inflammation and autoimmunity in both humans and mice (102–104). Thus, the multiple actions of SPMs on innate and adaptive immunity provide novel opportunities for SPM-based biomarker and drug development (**Figure 3**). The production of metabolically stable SPM analogs or receptor agonist with increased half-life, together with the design of nanocarriers to protect SPMs from degradation, may accelerate the way toward clinical translation.

## Author Contributions

JP-H, VC, SP, and SY wrote and revised the manuscript. All authors contributed to the article and approved the submitted version.

## Funding

The laboratory of SY and JP-H is supported by grants from the EFSD/JDRF/Lilly European Programme in Type 1 Diabetes Research 2019, the *Agence Nationale de la Recherche* (ANR-17-CE17-0004, ANR-18-IDEX-0001), the *Fondation pour la Recherche Médicale* (EQU20193007831), the *Association des Jeunes Diabétiques*, and the Innovative Medicines Initiative 2 Joint Undertaking under grant agreements 115797 and 945268 (INNODIA and INNODIA HARVEST), which receive support from the EU Horizon 2020 program, the European Federation of Pharmaceutical Industries and Associations, JDRF, and The Leona M. and Harry B. Helmsley Charitable Trust. VC is funded by the Italian Ministry of Health (grant GR-2016-02362380), the Italian Foundation of Multiple Sclerosis (FISM 2017/R/08), and the MAI Award Grant (MAI-VC-2021). SP is funded by the *Conseil Régional de Franche-Comté* (AAP 2019), the *Agence Nationale de la Recherche* (ANR-11-LABX-0021 and ANR-17-CE17-0004), BPI France (grant No. DOS0060162/00), and the European Union through the European Regional Development Fund of the Région Bourgogne-Franche-Comte (FC0013440).

## Conflict of Interest

SP is the CEO and shareholder of MED’INN’Pharma, which develops pro-resolutive drug candidates.

The remaining authors declare that the research was conducted in the absence of any commercial or financial relationships that could be construed as a potential conflict of interest.

## Publisher’s Note

All claims expressed in this article are solely those of the authors and do not necessarily represent those of their affiliated organizations, or those of the publisher, the editors and the reviewers. Any product that may be evaluated in this article, or claim that may be made by its manufacturer, is not guaranteed or endorsed by the publisher.
